# Chemical Synthesis of Marine-Derived Sulfoglycolipids, a New Class of Molecular Adjuvants

**DOI:** 10.3390/md15090288

**Published:** 2017-09-20

**Authors:** Emiliano Manzo, Laura Fioretto, Dario Pagano, Genoveffa Nuzzo, Carmela Gallo, Raffaele De Palma, Angelo Fontana

**Affiliations:** 1 Bio-Organic Chemistry Unit, CNR-Institute of Biomolecular Chemistry, Via Campi Flegrei 34, IT-80078 Pozzuoli, 80078 Napoli, Italy; laura_fioretto@hotmail.it (L.F.); dariopagano85@gmail.com (D.P.); nuzzo.genoveffa@icb.cnr.it (G.N.); carmen.gallo@icb.cnr.it (C.G.); afontana@icb.cnr.it (A.F.); 2 Department of Internal and Experimental Clinic, Clinical Immunology and Allergology, University of Campania, c/o II Policlinico (Bd. 3), Via S.Pansini 5, 80131 Napoli, Italy; Raffaele.DEPALMA@unicampania.it

**Keywords:** glycolipids, sulfolipid, vaccine, adjuvant, immunology, innate immunity, dendritic cells, immunogenic lipid, Sulfavant A

## Abstract

Vaccines play a primary role in the protection of human health by preventing infectious and chronic diseases. Recently we have reported 1,2-*O*-distearoyl-3-*O*-β-d-sulfoquinovosylglycerol (β-SQDG18), here named Sulfavant A (**1**), which shows promising properties as a new molecular adjuvant in in vitro and in vivo tests. In the present manuscript, we provide full details about a synthetic strategy for the preparation of **1**, including a discussion of chemical determinants of the activity and the major technical hurdles we faced during the study. Synthesis of Sulfavant A (**1**) is achieved by a versatile procedure based on a trichloroacetimidate methodology and peracetate sugar precursors. The final design opens possibilities for the preparation of a series of interesting analogs for further pharmacological optimization and development, including derivatives containing different saturated and polyunsaturated fatty acids (e.g., **17** and **22**).

## 1. Introduction

Vaccination consists of the stimulation of immune response by vaccines, historically composed of attenuated or inactivated biological agents [1]. However, most recent vaccines are constituted by peptides or recombinant DNA, which are safer, but often insufficiently immunogenic [1]. To overcome this problem, modern vaccines contain adjuvants that are necessary to boost the immune response. To date, except for the monophosphoryl lipid A (MPLA), clinically approved formulations of adjuvants are restricted to aluminum salts and emulsions of lipids in water [2].

Dendritic cells (DCs) are the most important antigen-presenting cells (APCs), and play a pivotal role in starting immunogenic reactions and priming a long-lasting and antigen-specific response [3]. The ability to activate DCs is a key tool for improving the efficacy of vaccination [3].

**Figure 1:**
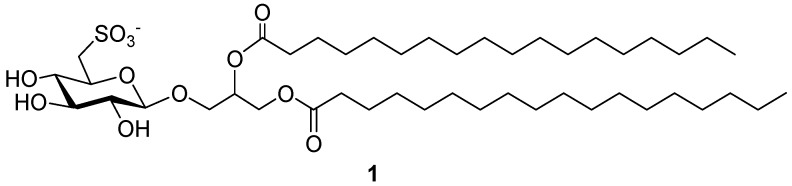
1,2-*O*-distearoyl-β-6′-sulfoquinovosylglycerol (Sulfavant A).

Recently, in our ongoing research on immunomodulatory substances, we reported the pharmacological development of the non-natural 1,2-*O*-distearoyl-3-*O*-β-d-sulfoquinovosylglycerol, here named Sulfavant A (**1**) (Figure 1), as a prototype for a new class of molecular adjuvants based on a sulfolipid skeleton [4]. This family of products is structurally inspired by the natural α-d-sulfoquinovosyl-diacylglycerols (α-SQDG), bioactive metabolites bearing a 6-sulfonic function on d-glucose (sulfoquinovose) linked to a glycerol moiety with two fatty acyl chains [5,6,7,8,9,10,11,12,13,14,15,16,17,18,19,20]. The natural sulfoglycoglycerolipids are marked by the α-configuration of the anomeric carbon and the β-configuration type is not present in nature.

Sulfavant A (**1**) activates human DCs and triggers an efficient immune response in vivo [4]. After treatment, DCs show up-regulation of MHC II molecules and other co-stimulatory proteins (CD83, CD86), as well as production of pro-inflammatory cytokines (IL-12 and INF-γ). Mice immunized with OVA associated to Sulfavant A produced a release of anti-OVA Ig comparable to traditional adjuvants, while, in an experimental model of melanoma, vaccination of C57BL/6 mice with Sulfavant A-adjuvanted hgp10 peptide elicited a protective response leading to a promising reduction in tumor growth and increase of mice survival [4].

Here we discuss in detail the strategy for the synthesis of Sulfavant A (**1**), including an overview of the main technical hurdles we have faced, together with the considerations for the scale-up of the process and the possibility to expand the class of synthesizable analogs.

## 2. Results and Discussion

**Scheme 1:**
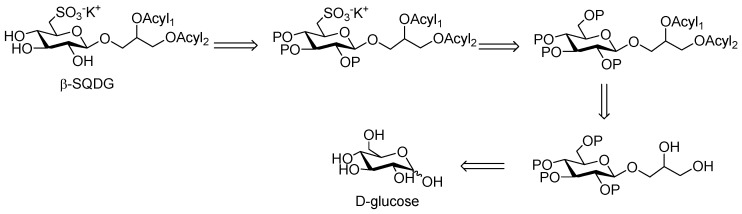
Retrosynthetic scheme for preparation of Sulfavant A (**1**) and its analogs.

For the preparation of Sulfavant A, the main challenges lay in the requirement of planning a chemical strategy comprising the introduction of the β-glycosidic bond at the anomeric carbon and the insertion of the sulfonic function on C_6_ of glucose (Scheme 1). At the same time, the possibility of introducing different and unsaturated fatty acyl residues on the glycerol moiety would make the strategy expandable to a wide class of analogs. Furthermore, the process had to be suitable to scale-up for further pharmacological development.

The first crucial point was the choice of orthogonal protecting groups on sugar, whose deprotection should not affect the diacylglycerol aglycon part and the glycosidic linkage. In this frame, tert-butyldimethylsilyl and benzyl groups to which the synthesis of α- and β-SQDGs [14,21,22] had already been applied, were initially tested for the preparation of the sulfolipid. Unfortunately, the tert-butyldimethylsilyl group proved to be too labile in the experimental conditions and this, together with its low deprotection efficiency, led to a considerable decrease in yields; whereas the use of benzyl excluded the possibility of introducing unsaturated acyl chains to the glycerol, and greatly reduced the possibility of extending the synthesis to other analogs. For these reasons, we decided to explore the use of acetate as transient protecting group for the hydroxy functions of the sugar moiety. The acetoxy derivatives are stable under a wide range of conditions and, most importantly, the presence of the acetate residue at C-2′ is capable of ensuring an excellent stereoselectivity in the formation of the β glycosidic bond by anchimeric assistance [23,24]. On the other hand, acetate removal implies basic conditions that are not suitable for preserving the ester linkage of glycerol with the acyl chains. To this end, a careful tuning of hydrolysis by hydrazine was crucial to completing the synthesis of the sulfolipid [23,24].

**Scheme 2:**
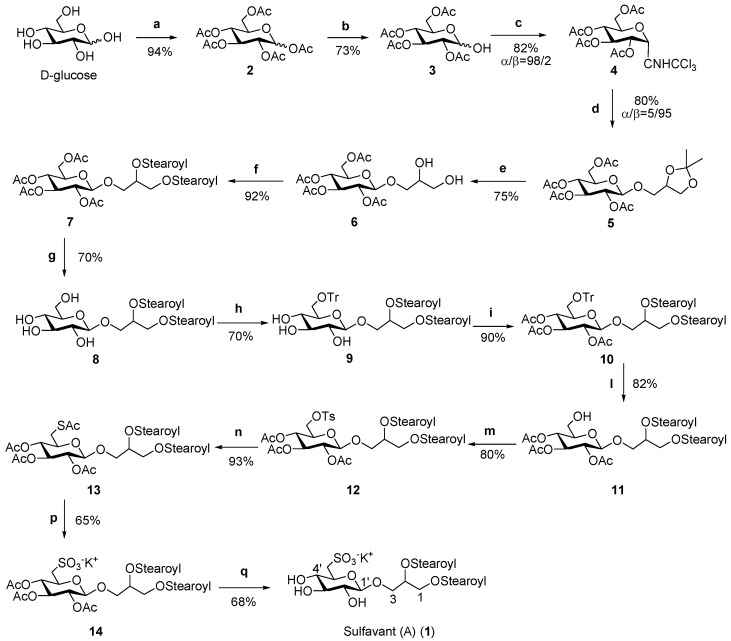
(**a**) Ac_2_O/pyridine; (**b**) BnNH_2_, THF; (**c**) Trichloroacetonitrile, DBU, dichloromethane, 0 °C; (**d**) BF_3_-Et_2_O, glycerol acetonide, dichloromethane, molecular sieves 4A, −10 °C; (**e**) Zn(NO_3_)_6_-6H_2_O, acetonitrile, 50 °C; (**f**) Stearic acid, DCC, DMAP, dichloromethane; (**g**) hydrazine mono-hydrate, EtOH 85%, 45 °C; (**h**) TrCl, pyridine; (**i**) Ac_2_O/pyridine; (**l**) I_2_-MeOH (1%), 60 °C; (**m**) Tosyl chloride, pyridine; (**n**) Potassium thioacetate, 2 butenone, 80 °C; (**p**) Hydrogen peroxide, acetic acid, potassium acetate, 40 °C; (**q**) hydrazine mono-hydrate, EtOH 85%, 44 °C.

As reported in Scheme 2, the total synthesis of Sulfavant A (**1**) started with acetylation of d-glucose, followed by selective deacetylation at the anomeric position with benzylamine and derivatization with trichloroacetimidate. The subsequent coupling with 1,2-*O*-isopropylidene glycerol by Schmidt methodology [25,26] gave the derivative **5** in high yields (about 80%). As expected, the acetate groups determined the high stereoselectivity of the reaction (β/α ratio 95:5) with the β-orientation of the glycosidic bond by neighboring group participation.

The next step was the removal of the isopropylidene residue under soft acidic conditions to preserve the glycosidic linkage. Use of zinc nitrate hexahydrate in acetonitrile allowed hydrolysis in quantitative yields [27], whereas *N*,*N*-dicyclohexylcarbodiimide (DCC)-mediated condensation with two equivalents of stearic acid yielded the diacyl derivative **7**. At this point, with the aim of introducing the sulfonic function on carbon 6′, a selective removal of sugar acetyl groups without affecting the acyls on glycerol was necessary to obtain β-glucosyl-distearoyl-glycerol **8**. This was achieved by an accurate setting of the basic conditions that drove us to use monohydrate hydrazine in an amount of 2.4 moles per acetate unit at a temperature below 45 °C [23,24]. After trytilation of the primary alcohol of glucose (compound **9**, 70 %), acetylation (compound **10**, 89%), detrytilation [28] (compound **11**, 82%) and subsequent introduction on C-6′ of the tosyl function (compound **12**, 80%) permitted the insertion of a thioacetate group (compound **13**, 93%) [29]. Final oxidation by hydrogen peroxide gave the corresponding sulfonic acid (compound **14**, 65%), which was fully deacetylated by monohydrate hydrazine to give Sulfavant A (**1**) (68%), whose NMR data are reported in Table 1.

Synthesis of Sulfavant A (**1**) was characterized by clean and efficient steps, along with a simple and easy workup, which makes the procedure suited for subsequent and crucial scaling-up. In fact, we did not face any problems in the transition from small to larger volumes. Furthermore, the workup did not require any particular experimental change in order to prepare hundreds of milligrams (400 mg) of the product. On the basis of this process, we also expect that the synthetic procedure is able to provide grams of the vaccine adjuvant for in vivo tests and future pharmacological development.

**Scheme 3:**
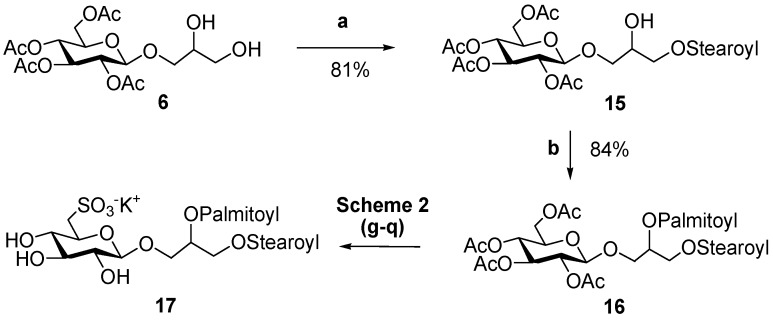
(**a**) Stearic acid (1 equiv.), DCC, DMAP, dichloromethane, 4 °C. (**b**) Palmitic acid (1,2 equiv.), DCC, DMAP, dichloromethane.

The design of the synthetic strategy also opens possibilities for the preparation of a large range of analogs, including compounds with mixed composition of fatty acids or unsaturated acyl chains on glycerol moiety. An example of this is reported in Scheme 3 for the synthesis of **17**, 1-*O*-stearoyl-2-*O*-palmitoyl-3-*O*-β-d-sulfoquinovosylglycerol, which contains two different fatty acids resgiospecifically distributed at the two positions of glycerol.

**Figure 2:**
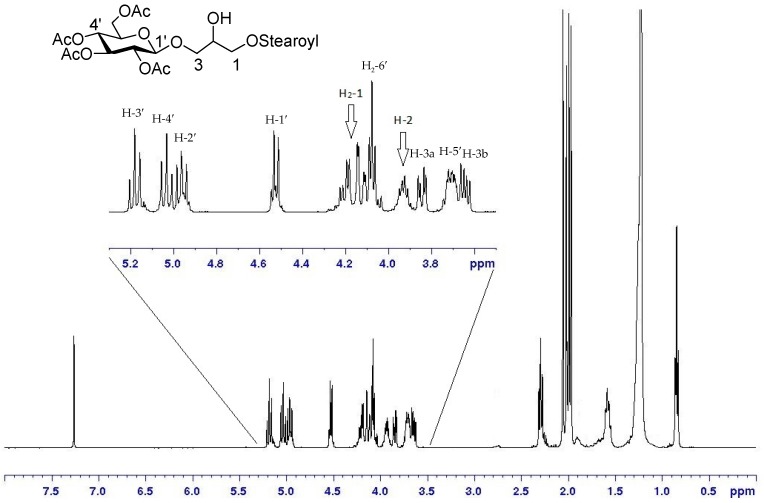
^1^H-NMR of **15** (CDCl_3_, 400MHz) and enlargement of the area between 5.3 and 3.5 ppm.

In this case, use of DCC at 4 °C permitted a good control of the step-wise introduction of two different acyl chains and gave yields of the monoacyl derivative **15** (81%) significantly higher than those obtained by other methods [30,31,32]. Acylation of the primary hydroxy residue was largely favored in the first coupling reaction with equimolar amounts of fatty acid, and we did not observe any byproduct at C-2 of **15**, as indicated by the NMR spectra, which showed deshielding of H_2_-1 signals at δ_H_ 4.21–4.11 (long range correlation of H_2_-1 to a carboxylic ester at δ_C_ 171.1) and δ_H_ value of H-2, linked to non-acylated oxygen, at 3.91 ppm (Figure 2).

**Scheme 4:**
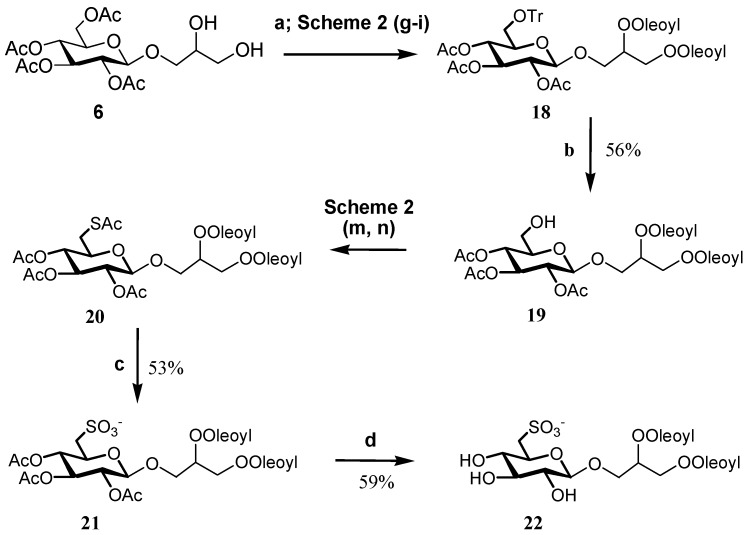
(a) Oleic acid (2.3 equiv.), DCC, DMAP, dichloromethane; (b) Dowex-H^+^, MeOH/H_2_O (95/5), 50 °C; (c) (NH_4_)_6_Mo_7_O_24_-4H_2_O, H_2_O_2_, MeOH, 50 °C; (d) hydrazine mono-hydrate (2.4 equiv.), EtOH 85%.

The introduction of acetate as protecting group also offers access to unsaturated analogs, as long as it is possible to control the sensitivity of double bonds to both the iodine and the hydrogen peroxide/acetic acid/acetate systems that are used for removal of the trityl group and sulfur oxidation, respectively. As shown in Scheme 4, this could be achieved by a slight modification of the synthetic procedure, and the use of an acidic resin to deprotect the primary hydroxy group and molybdate oxidation for the final step. Soft acidic conditions by Dowex H^+^ at 50 °C in methanol/water 95/5 allowed a complete removal of the trityl group while preserving the glycosidic bond of **19**. The reaction led to formation of a small amount of other hydrolyzed byproducts, which were removed by chromatographic purification, but resulted in a slight decrease in the synthetic yield in comparison with the original step used for Sulfavant A. Molybdate oxidation has been used already for synthesis of α-SQDGs [14], but application in the preparation of the β-SQDG required a careful adjustment of the reaction time. Increase in the temperature from room value to 50 °C, together with the use of twice the amount of ammonium heptamolybdate tetrahydrate and hydrogen peroxide, favoured the course of the reaction and led to the dioleoyl analog **22** with yield close to 60%.

## 3. Materials and Methods

1D- and 2D-NMR spectra were recorded on a Bruker Avance-400 (400.13 MHz) and on a Bruker DRX-600 equipped with a TXI CryoProbe in CDCl_3_ and CD_3_OD (δ values are referred to CHCl_3_ and CH_3_OH at 7.26 and 3.34 ppm respectively) and ^13^C NMR spectra were recorded on a Bruker DPX-300 (75.47 MHz) (δ values are referred to CDCl_3_ and CD_3_OD at 77.0 and 49.0 ppm respectively). HRESIMS was conducted on a Micromass Q-TOF micro. TLC plates (Kieselgel 60 F_254_) and silica gel powder (Kieselgel 60, 0.063–0.200 mm) were from Merck.

All the reagents were purchased from Sigma-Aldrich and used without any further purification.

### Preparation and Characterization of Synthetic Molecules

All the experimental procedures and spectral data of Sulfavant A (**1**) and synthetic intermediates **2**–**14** were reported in reference 4.

**Compound 15**: compound **6** (0.148 g, 0.00035 mol) was dissolved in anhydrous dichloromethane (5 mL); stearic acid (0.0994 g, 0.00035 mol), dicyclohexylcarbodiimide (0.0721 g, 0.00035 mol) and DMAP (0.00427 g, 0.000035 mol) were added at 0 °C and the reaction mixture was stirred overnight at 4 °C; after evaporation under reduced pressure, the mixture was purified by silica gel chromatography using a gradient of petroleum ether/diethyl ether to give compound **15** (0.165 g, 0.000284 mol, 81%). ^1^H-NMR (400 MHz, CDCl3): δ 5.18 (1H, t, *J* = 9.6 Hz, H-3′), 5.04 (1H, t, *J* = 9.6 Hz, H-4′), 4.97 (1H, dd, *J* = 8.1, 9.6 Hz, H-2′), 4.53 and 4.51 (each for 1H of the two epimers, d, *J* = 8.1 Hz, H-1′), 4.20 (1H, dd, *J* = 5.2, 12.0 Hz, H-1a), 4.13 (1H, dd, *J* = 2.4, 12.0 Hz, H-1b), 4.09–4.06 (2H, m, H_2_-6′), 3.91(1H, m, H-2), 3.84 (1H, dd, 3.4, 10.3 Hz, H-3a), 3.70, (1H, m, H-5′), 3.64 (1H, dd, 5.7, 10.3 Hz, H-3b), 2.29 (2H, t, *J* = 7.1 Hz, α-methylene ), 2.07–1.96 (12H, s, 4 OAc), 1.61–1.52 (2H, m, β-methylene), 1.30–1.18 (acyl chain methylenes), 0.84 (3H, t, 6.8 Hz, CH_3_); HRESIMS *m*/*z* 711.3940 [M + Na]+ (calcd for C_35_H_60_O_13_Na, 711.3932).

**Compound 16**: compound **15** (0.165 g, 0.000284 mol) was dissolved in anhydrous dichloromethane (6 mL); palmitic acid (0.0738 g, 0.00034 mol), dicyclohexylcarbodiimide (0.07 g, 0.00034 mol) and DMAP (0.0042 g, 0.000034 mol) were added and the reaction mixture was stirred overnight; after evaporation under reduced pressure, the mixture was purified by silica gel chromatography using a gradient of petroleum ether/diethyl ether to give compound **16** (0.228 g, 0.00024 mol, 84%); NMR data were identical to those of **7** [4]; HRESIMS *m*/*z* 949.6235 [M + Na]^+^ (calcd for C_51_H_90_O_14_Na, 949.6228).

**Compound 17**: NMR data were identical to those of Sulfavant A (**1**) (Table 1); HRESIMS *m*/*z* 821.5454 [M − K]^−^ (calcd for C_43_H_81_O_12_S^−^, 821.5454).

**Compound 18**: ^1^H-NMR (400 MHz, CDCl_3_): δ 7.47–7.18 (15H, m, trityl portion), 5.36–5.32 (4H, m, olefinic protons), 5.23 (1H, m, H-2), 5.15 (1H, m, H-4′), 5.14 (1H, m, H-3′), 5.05 (1H, m, H-2′), 4.54 (1H, d, *J* = 7.7 Hz, H-1′), 4.34 (1H, dd, *J* = 4.1, 12.0 Hz, H-1a), 4.13 (1H, dd, *J* = 5.9, 12.0 Hz, H-1b), 4.03 (1H, dd, *J* = 4.9, 11.1 Hz, H-3a), 3.74 (1H, dd, *J* = 5.1, 11.1 Hz, H-3b), 3.55 (1H, m, H-5′), 3.27 (1H, bd, *J* = 10.2 Hz, H-6′a), 3.09 (1H, dd, *J* = 2.3, 10.2, H-6′b), 2.31 (2H, t, *J* = 7.5 Hz, α-methylene), 2.16 (2H, t, *J* = 7.8 Hz, α-methylene), 2.10–2.01 (9H, s, 3 OAc), 2.03–2.00 (8H, overlapped, allylic protons), 1.54 (4H, m, β-methylene), 1.28–1.15 (acyl chain), 0.86 (6H, t, *J* = 7.0 Hz, 2CH_3_); HRESIMS *m*/*z* 1173.7224 [M + Na]^+^ (calcd for C_70_H_102_O_13_Na, 1173.7218).

**Compound 19**: compound **18** (0.1 g, 0.000087 mol) was dissolved in methanol/water (95/5) solution and dowex-H^+^ (1.1 g) was added and the reaction was stirred at 50 °C overnight; after filtration and evaporation under reduced pressure, the reaction mixture was purified by silica gel chromatography using a gradient of petroleum ether/diethyl ether to give compound **19** (0.044 g, 0.000048, 56%); δ 5.37–5.33 (4H, m, olefinic protons), 5.31–5.28 (2H, overlapped, H-2, H-3′), 5.08–5.00 (2H, m, H-2′, H-4′), 4.61 (1H, d, *J* = 7.8 Hz, H-1′), 4.36 (1H, m, H-1a), 4.16 (1H, m, H-1b), 3.92 (1H, m, H-3a), 3.78 (1H, m, H-3b), 3.74 (1H, m, H-6′a), 3.60 (1H, m, H-6′b), 3.54 (1H, m, H-5′), 2.41–2.29 (4H, m, α-methylene), 2.10–2.01 (9H, s, 3 OAc), 2.04–2.01 (8H, overlapped, allylic protons), 1.62–1.52 (4H, m, β-methylene), 1.33–1.15 (acyl chain), 0.90 (6H, t, *J* = 7.0 Hz, 2CH_3_); HRESIMS *m*/*z* 931.6118 [M + Na]^+^ (calcd for C_51_H_88_O_13_Na, 931.6123).

**Compound 20**: ^1^H-NMR (400 MHz, CDCl_3_): δ 5.35–5.31 (4H, m, olefinic protons), 5.18–5.14 (2H, m, H-2, H-3′), 4.96–4.89 (2H, m, H-2′, H-4′), 4.50 (1H, d, *J* = 8.0 Hz, H-1′), 4.28 (1H, dd, *J* = 4.1, 11.8 Hz, H-1a), 4.08 (1H, dd, *J* = 5.7, 11.8 Hz, H-1b), 3.91 (1H, dd, *J* = 4.5, 11.1 Hz, H-3a), 3.65 (1H, dd, *J* = 5.4, 11.1 Hz, H-3b), 3.62 (1H, m, H-5′), 3.24 (1H, bd, *J* = 11.4 Hz, H-6′a), 3.05 (1H, dd, *J* = 2.4 Hz, 11.4 Hz), 2.33–2.29 (4H, m, α-methylene), 2.13–1.98 (9H, s, 3 OAc), 2.05–2.01 (8H, overlapped, allylic protons), 1.64–1.57 (4H, m, β-methylene), 1.32–1.23 (acyl chain), 0.91–0.87 (6H, overlapped, 2CH_3_); HRESIMS m/z 989.6007 [M + Na]^+^ (calcd for C_53_H_90_O_13_NaS, 989.6000).

**Compound 21**: to a solution of compound **20** (0.04 g, 0.000041 mol) in methanol (8 mL) ammonium heptamolybdate tetrahydrate [(NH_4_)_6_Mo_7_O_24_4H_2_O] (0.051 g, 1 equiv., 0.000041 mol) in 30% H_2_O_2_ (1.0 mL) was added with stirring. After 24 h at 50 °C the mixture was diluted with water and extracted with ethylacetate; the organic phase was purified by silica gel chromatography using a gradient of chloroform/methanol to give **21** (0.022 g, 0.000022 mol, 53%); δ 5.36–5.31 (4 H, m, olefinic protons), 5.27 (1H, m, H-2), 5.18 (1H, dd, 8.9, 8.9 Hz, H-3), 5.08–4.99 (2H, m, H-2′, H-4′), 4.68 (1H, d, 7.3 Hz, H-1′), 4.31 (1H, dd, *J* = 5.1, 11.1 Hz, H-1a), 4.15–4.06 (3H, overlapped, H-1b, H-3a, H-5′), 3.75 (1H, m, H-3b), 3.20 (2H, overlapped, H_2_-6′), 2.33–2.28 (4H, m, α-methylene), 2.06–1.98 (9H, s, 3 OAc), 2.05–2.00 (8H, overlapped, allylic protons), 1.64–1.55 (4H, m, β-methylene), 1.34–1.22 (acyl chain), 0.91–0.88 (6H, overlapped, 2CH_3_); HRESIMS *m*/*z* 971.5764 [M − K]^−^ (calcd for C_51_H_87_O_15_S^−^, 971.5771).

**Compound 22**: compound **21** (0.022 g, 0.000022 mol) was dissolved in aq. ethanol (85%) (4 mL), hydrazine monohydrate (0.00016 mol, 2.4 equiv.) was added, and the reaction mixture was stirred for 6 h at 44 °C. After evaporation under a stream of nitrogen, the mixture was purified by silica gel chromatography using a gradient of chloroform/methanol to obtain **22** (0.011 g, 0.000013 mol, 59%) ^1^H-NMR (400 MHz, CDCl_3_/CD_3_OD 1/1): δ 5.34–5.27 (5H, overlapped, H-2 and olefinic protons), 4.47 (1H, m, H-1a), 4.34 and 4.32 (each for 1H, d, 7.8 Hz, H-1′), 4.19 (1H, m, H-1b), 4.13–4.03 (1H, m, H-1a), 3.79–3.75 (2H, m, H-3b, H-5′), 3.42 (1H, m, H-3′), 3.32 (1H, m, H-6′a), 3.26 (1H, m, H-2′), 3.14 (1H, m, H-4′), 2.98 (1H, m, H-6′b), 2.43–2.35 (4H, m, α-methylene), 2.06–2.01 (8H, m, allylic protons), 1.69–1.58 (4H, m, β-methylene), 1.43–1.29 (acyl chain), 0.94 (6H, overlapped, 2CH_3_); HRESIMS *m*/*z* 845.5460 [M − K]^−^ (calcd for C_45_H_81_O_12_S^−^, 845.5454).

## 4. Conclusions

Sulfavant A (**1**) is a synthetic β-sulfoglycolipid with promising potential for immunomodulant and adjuvant activity. Synthesis of this molecule was achieved by a versatile and simple chemical procedure involving stereoselective glycosylation of trichloroacetimidate-glucose donor with 1,2-*O*-isopropylidene glycerol acceptor. In addition to the simplification of the synthetic steps, the trichloroacetimidate methodology and the use of acetate as the main protecting group of the sugar donor permit the synthesis in high yields of saturated and unsaturated β-anomer SQDG derivatives with mixed composition of fatty acids (e.g., **17** and **22**). The proposed procedure has versatile applications, and allows access to a wide family of regio-pure compounds. The efficiency and simplicity of the strategy also allow a scale-up of the synthetic approach, leading to amounts of pure β-SQDGs for in vivo tests and future pharmacological development.

## Figures and Tables

**Table 1:** ^1^H and ^13^C NMR data of Sulfavant A (1) in CDCl_3_:CD_3_OD (1:1), 400 MHz.

Position	δ_C_	δ_H_, m, *J* (Hz)
1′	103.4	4.34/4.32 (each for 1H), d, 7.8
2′	73.6	3.26, m
3′	76.5	3.42, m
4′	73.1	3.14, m
5′	72.5	3.79–3.75, m
6′a	53.8	3.32, m
6′b	53.8	2.98, m
1a	63.3	4.47, m
1b	63.3	4.19, m
2	70.5	5.29, m
3a	67.8	4.13–4.03, m
3b	67.8	3.79–3.75, m
Fatty acyls		
**α-CH_2_**	34.4	2.43–2.35, overlapped
**β-CH_2_**	25.1	1.69–1.54, overlapped
**CH_2_ (chain)**	30.4–29.6	1.32–1.25, overlapped
**CH_3_**	14.1	0.94, overlapped

## Supplementary Materials

The following are available online at http://www.mdpi.com/1660-3397/15/9/288/s1, Figure S1: COSY and HMBC spectra of 15.
