# Supplementary material for: Chemical Synthesis of Marine-Derived Sulfoglycolipids, a New Class of Molecular Adjuvants
Source: Mar Drugs. 2017 Sep 20;15(9):288. doi: 10.3390/md15090288 (PMC5618427; doi:10.3390/md15090288)

Supporting Information

Chemical synthesis of marine-derived sulfoglycolipids, a new class of molecular adjuvants

Emiliano Manzo[1,*], Laura Fioretto[1], Dario Pagano[1], Genoveffa Nuzzo[1], Carmela Gallo[1], Raffaele De Palma[2] and Angelo Fontana[1]

[1] Bio-Organic Chemistry Unit, CNR- Institute of Biomolecular Chemistry, Via Campi Flegrei 34, IT-80078 Pozzuoli, Napoli, Italy

[2] University of Campania, Clinical Immunology and Allergology, Dept. of Internal and Experimental Clinic, c/o II Policlinico (Bd. 3), Via S.Pansini, 5, 80131 Napoli, Italy

COSY and HMBC spectra of **15**

^{1}H-^{1}H COSY spectrum of **15** ($CDCl_3$, 400 MHz)

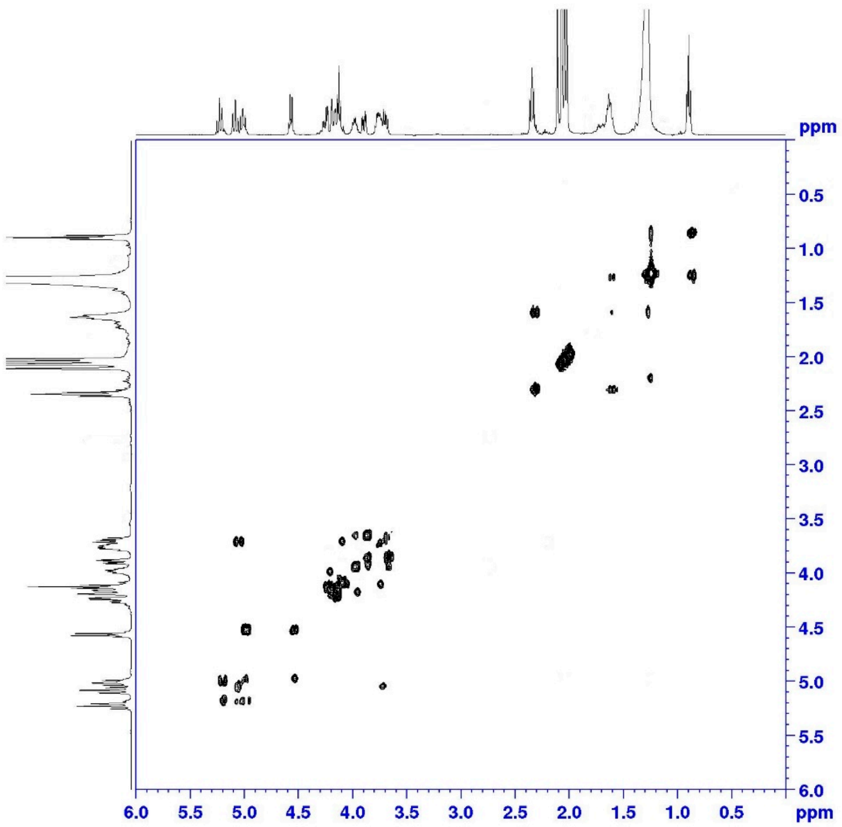

HMBC spectrum of **15** ($CDCl_3$, 400 MHz)

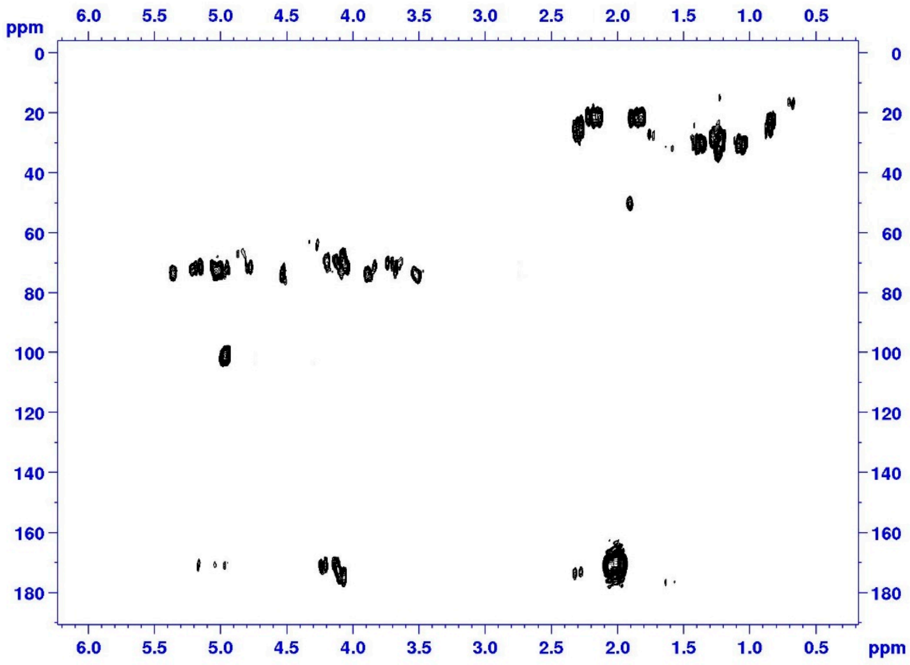

Enlargement of HMBC spectrum of **15** ($CDCl_3$, 400 MHz)

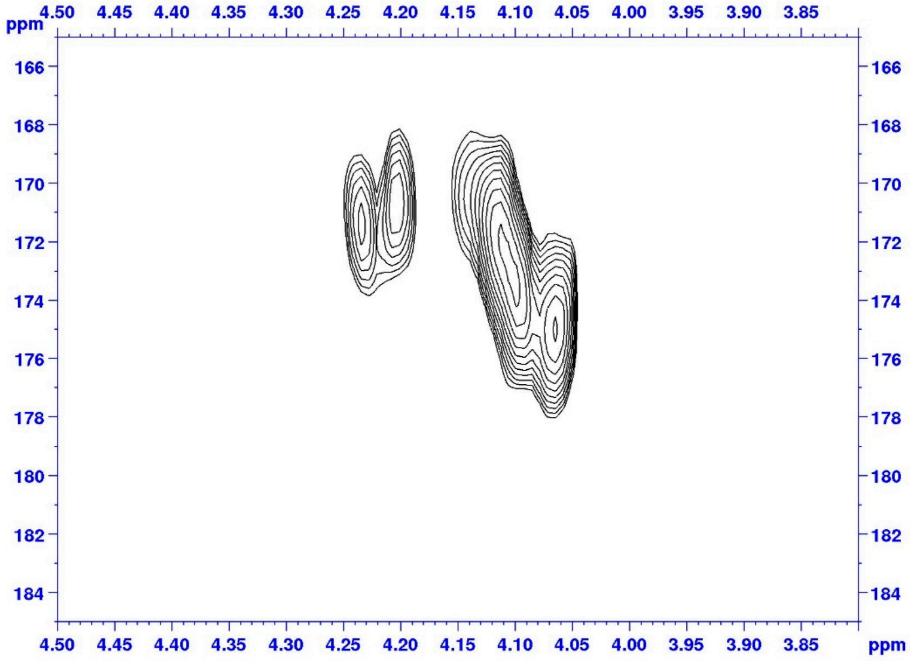

Supplement: Supplementary file 1 [file marinedrugs-15-00288-s001.pdf]
